# Supplementary material for: The paradoxical effect of oral prednisone in granulomatous lobular mastitis: An observational cohort study
Source: PLoS One. 2026 Apr 8;21(4):e0341901. doi: 10.1371/journal.pone.0341901 (PMC13061257; doi:10.1371/journal.pone.0341901)
Supplement: S1 Table — (DOCX) [file pone.0341901.s001.docx]

**S1 Table. Univariable and multivariable Cox regression analysis restricted to the prospective cohort (n=374).**

| Factors | N(%)/x ± s | Univariate | Multivariate | |
| --- | --- | --- | --- | --- |
|  |  | P value | HR(95%CI) | P value |
| Age at diagnosis (year) | 31.7 ± 4.7 | 0.323 | 0.970(0.917-1.025) | 0.282 |
| Weight (kg) | 61.5 ± 10.8 | 0.533 | 1.007(0.986-1.030) | 0.508 |
| Affected side |  | 0.007 |  | <0.001 |
| left | 194(51.9) |  | Ref |  |
| right | 170(45.5) |  | 1.693(1.025-2.798) | 0.040 |
| bilateral | 10(2.7) |  | 9.535(3.059-29.722) | <0.001 |
| Days to first visit (days) | 18.5 ± 29.6 | 0.433 | 0.989(0.977-1.002) | 0.086 |
| Maximum lesion diameter on ultrasound |  | 0.022 |  | 0.152 |
| < 4 cm | 142(38.0) |  | Ref |  |
| ≥ 4 cm | 225(60.2) |  | 0.899(0.542-1.490) | 0.680 |
| unknown | 7(1.9) |  | 0.051(0.003-1.003) | 0.053 |
| Ultrasound-detected lesion count |  | <0.001 |  | 0.029 |
| solitary | 52(13.9) |  | Ref |  |
| multiple | 314(84.0) |  | 0.839(0.400-1.761) | 0.643 |
| unknown | 8(2.1) |  | 16.487(1.653-164.416) | 0.017 |
| Mammary abscess |  | 0.723 |  | 0.149 |
| no | 50(13.4) |  | Ref |  |
| yes | 324(86.6) |  | 1.812(0.809-4.059) |  |
| Microabscess |  | 0.003 |  | 0.058 |
| no | 170(45.5) |  | Ref |  |
| yes | 197(52.7) |  | 0.572(0.350-0.935) | 0.026 |
| unknown | 7(1.9) |  | 1.972(0.294-13.245) | 0.485 |
| White blood cell |  | <0.001 |  | 0.001 |
| < 10*10^9/L | 199(53.2) |  | Ref |  |
| ≥ 10*10^9/L | 170(45.5) |  | 1.825(1.046-3.184) | 0.034 |
| unknown | 5(1.3) |  | 17.661(3.421-91.159) | 0.001 |
| C-reactive protein |  | 0.007 |  | 0.194 |
| < 10 mg/L | 171(45.7) |  | Ref |  |
| ≥ 10 mg/L | 122(32.6) |  | 0.913(0.480-1.738) | 0.782 |
| unknown | 81(21.7) |  | 1.643(0.872-3.096) | 0.125 |
| Prolactin |  | 0.004 |  | 0.474 |
| ≤ 650 uIU/ml | 256(68.4) |  | Ref |  |
| > 650 uIU/ml | 101(27.0) |  | 1.317(0.723-2.402) | 0.368 |
| unknown | 17(4.5) |  | 1.762(0.475-6.538) | 0.397 |
| Hyperlipidemia |  | 0.872 |  | 0.712 |
| no | 142(38.0) |  | Ref |  |
| yes | 138(36.9) |  | 0.996(0.582-1.704) | 0.988 |
| unknown | 94(25.1) |  | 0.741(0.352-1.563) | 0.431 |
| Quinolone therapy |  | 0.243 |  | 0.945 |
| no | 38(10.2) |  | Ref |  |
| yes | 336(89.8) |  | 0.972(0.439-2.151) |  |
| Penicillin therapy |  | 0.367 |  | 0.317 |
| no | 356(95.2) |  | Ref |  |
| yes | 18(4.8) |  | 0.450(0.095-2.147) |  |
| Cephalosporin therapy |  | 0.833 |  | 0.225 |
| no | 276(73.8) |  | Ref |  |
| yes | 98(26.2) |  | 1.408(0.810-2.448) |  |
| Macrolide therapy |  | 0.609 |  | 0.450 |
| no | 356(95.2) |  | Ref |  |
| yes | 18(4.8) |  | 1.514(0.517-4.435) |  |
| Nitroimidazole therapy |  | 0.130 |  | 0.032 |
| no | 363(97.1) |  | Ref |  |
| yes | 11(2.9) |  | 3.609(1.119-11.642) |  |
| Antitubercular therapy |  | 0.038 |  | 0.012 |
| no | 277(74.1) |  | Ref |  |
| yes | 97(25.9) |  | 0.433(0.225-0.833) |  |
| Oral prednisone |  | 0.001 |  | 0.005 |
| no | 133(35.6) |  | Ref |  |
| yes | 241(64.4) |  | 2.645(1.332-5.253) |  |
| Bromocriptine therapy |  | 0.626 |  | 0.577 |
| no | 181(48.4) |  | Ref |  |
| yes | 193(51.6) |  | 0.861(0.509-1.456) |  |
| Abscess drainage |  | 0.015 |  | 0.013 |
| no | 116(31.0) |  | Ref |  |
| yes | 258(69.0) |  | 0.439(0.229-0.839) |  |
| Surgical excision |  | 0.178 |  | 0.186 |
| no | 52(13.9) |  | Ref |  |
| yes | 322(86.1) |  | 0.587(0.267-1.292) |  |

Abbreviations: HR, hazard ratio; CI, confidence interval; Ref, reference. The normal reference range for serum prolactin is 80.56-650.84 uIU/ml.
