# Supplementary material for: The paradoxical effect of oral prednisone in granulomatous lobular mastitis: An observational cohort study
Source: PLoS One. 2026 Apr 8;21(4):e0341901. doi: 10.1371/journal.pone.0341901 (PMC13061257; doi:10.1371/journal.pone.0341901)
Supplement: S2 Table — (DOCX) [file pone.0341901.s002.docx]

**S2 Table. Baseline characteristics of patients before and after propensity score matching.**

| Factors | Before PSM | | | After PSM | | |
| --- | --- | --- | --- | --- | --- | --- |
|  | Non-prednisone group (n = 245) | Oral prednisone group (n = 369) | P value | Non-prednisone group (n = 171) | Oral prednisone group (n = 171) | P value |
|  | N(%)/x ± s | N(%)/x ± s |  | N(%)/x ± s | N(%)/x ± s |  |
| Age at diagnosis (year) | 31.2 ± 5.0 | 31.4 ± 4.7 | 0.581 | 31.6 ± 4.7 | 31.1 ± 4.6 | 0.365 |
| Weight (kg) | 60.1 ± 10.8 | 60.8 ± 9.9 | 0.392 | 61.1 ± 11.2 | 60.4 ± 10.0 | 0.545 |
| Affected side |  |  | 0.331 |  |  | 0.384 |
| left | 128(52.2) | 191(51.8) |  | 95(55.6) | 96(56.1) |  |
| right | 115(46.9) | 169(45.8) |  | 75(43.9) | 71(41.5) |  |
| bilateral | 2(0.8) | 9(2.4) |  | 1(0.6) | 4(2.3) |  |
| Days to first visit (days) | 20.6 ± 25.7 | 18.1 ± 30.2 | 0.287 | 19.2 ± 21.5 | 16.6 ± 21.8 | 0.279 |
| Maximum lesion diameter on ultrasound |  |  | 0.676 |  |  | 0.731 |
| < 4 cm | 91(37.1) | 148(40.1) |  | 58(33.9) | 65(38.0) |  |
| ≥ 4 cm | 137(55.9) | 200(54.2) |  | 102(59.6) | 96(56.1) |  |
| unknown | 17(6.9) | 21(5.7) |  | 11(6.4) | 10(5.8) |  |
| Ultrasound-detected lesion count |  |  | <0.001 |  |  | 0.590 |
| solitary | 58(23.7) | 39(10.6) |  | 31(18.1) | 25(14.6) |  |
| multiple | 170(69.4) | 308(83.5) |  | 127(74.3) | 135(78.9) |  |
| unknown | 17(6.9) | 22(6.0) |  | 13(7.6) | 11(6.4) |  |
| Mammary abscess |  |  | 0.006 |  |  | 1.000 |
| no | 28(11.4) | 73(19.8) |  | 23(13.5) | 23(13.5) |  |
| yes | 217(88.6) | 296(80.2) |  | 148(86.5) | 148(86.5) |  |
| Microabscess |  |  | <0.001 |  |  | 0.356 |
| no | 92(37.6) | 142(38.5) |  | 66(38.6) | 72(42.1) |  |
| yes | 77(31.4) | 166(45.0) |  | 62(36.3) | 67(39.2) |  |
| unknown | 76(31.0) | 61(16.5) |  | 43(25.1) | 32(18.7) |  |
| White blood cell |  |  | <0.001 |  |  | 0.139 |
| < 10*10^9/L | 169(69.0) | 167(45.3) |  | 110(64.3) | 125(73.1) |  |
| ≥ 10*10^9/L | 62(25.3) | 197(53.4) |  | 52(30.4) | 42(24.6) |  |
| unknown | 14(5.7) | 5(1.4) |  | 9(5.3) | 4(2.3) |  |
| C-reactive protein |  |  | 0.050 |  |  | 0.444 |
| < 10 mg/L | 89(36.3) | 122(33.1) |  | 66(38.6) | 55(32.2) |  |
| ≥ 10 mg/L | 49(20.0) | 106(28.7) |  | 39(22.8) | 41(24.0) |  |
| unknown | 107(43.7) | 141(38.2) |  | 66(38.6) | 75(43.9) |  |
| Prolactin |  |  | <0.001 |  |  | 0.790 |
| ≤ 650 uIU/ml | 141(57.6) | 250(67.8) |  | 104(60.8) | 110(64.3) |  |
| > 650 uIU/ml | 60(24.5) | 96(26.0) |  | 52(30.4) | 48(28.1) |  |
| unknown | 44(18.0) | 23(6.2) |  | 15(8.8) | 13(7.6) |  |
| Hyperlipidemia |  |  | 0.023 |  |  | 0.618 |
| no | 72(29.4) | 122(33.1) |  | 51(29.8) | 50(29.2) |  |
| yes | 67(27.3) | 127(34.4) |  | 52(30.4) | 60(35.1) |  |
| unknown | 106(43.3) | 120(32.5) |  | 68(39.8) | 61(35.7) |  |
| Quinolone therapy |  |  | <0.001 |  |  | 0.461 |
| no | 94(38.4) | 51(13.8) |  | 48(28.1) | 42(24.6) |  |
| yes | 151(61.6) | 318(86.2) |  | 123(71.9) | 129(75.4) |  |
| Penicillin therapy |  |  | 0.325 |  |  | 0.240 |
| no | 234(95.5) | 358(97.0) |  | 163(95.3) | 167(97.7) |  |
| yes | 11(4.5) | 11(3.0) |  | 8(4.7) | 4(2.3) |  |
| Cephalosporin therapy |  |  | 0.009 |  |  | 0.814 |
| no | 168(68.6) | 288(78.0) |  | 118(69.0) | 120(70.2) |  |
| yes | 77(31.4) | 81(22.0) |  | 53(31.0) | 51(29.8) |  |
| Macrolide therapy |  |  | 0.744 |  |  | 1.000 |
| no | 233(95.1) | 353(95.7) |  | 164(95.9) | 164(95.9) |  |
| yes | 12(4.9) | 16(4.3) |  | 7(4.1) | 7(4.1) |  |
| Nitroimidazole therapy |  |  | 0.207 |  |  | 0.396 |
| no | 232(94.7) | 357(96.7) |  | 163(95.3) | 166(97.1) |  |
| yes | 13(5.3) | 12(3.3) |  | 8(4.7) | 5(2.9) |  |
| Antitubercular therapy |  |  | 0.942 |  |  | 0.099 |
| no | 150(61.2) | 227(61.5) |  | 108(63.2) | 93(54.4) |  |
| yes | 95(38.8) | 142(38.5) |  | 63(36.8) | 78(45.6) |  |
| Bromocriptine therapy |  |  | 0.075 |  |  | 0.913 |
| no | 148(60.4) | 196(53.1) |  | 95(55.6) | 94(55.0) |  |
| yes | 97(39.6) | 173(46.9) |  | 76(44.4) | 77(45.0) |  |
| Abscess drainage |  |  | 0.004 |  |  | 0.903 |
| no | 58(23.7) | 128(34.7) |  | 45(26.3) | 46(26.9) |  |
| yes | 187(76.3) | 241(65.3) |  | 126(73.7) | 125(73.1) |  |
| Surgical excision |  |  | 0.016 |  |  | 0.784 |
| no | 63(25.7) | 65(17.6) |  | 32(18.7) | 34(19.9) |  |
| yes | 182(74.3) | 304(82.4) |  | 139(81.3) | 137(80.1) |  |
