# Supplementary material for: The paradoxical effect of oral prednisone in granulomatous lobular mastitis: An observational cohort study
Source: PLoS One. 2026 Apr 8;21(4):e0341901. doi: 10.1371/journal.pone.0341901 (PMC13061257; doi:10.1371/journal.pone.0341901)
Supplement: Attachment — Submitted filename: Response to Reviewers.docx [file pone.0341901.s005.docx]

Response to Reviewer Comments

Dear Reviewers,

Thank you very much for your valuable comments and suggestions on our study. We have carefully considered each of your points and have made corresponding improvements and supplements in the revised manuscript. Below is our detailed response to each of your comments:

Reviewer #1:

**Comment: The study is well designed and it will help to physicians to use oral steroid therapy for the patients with IGM. In fact I use intralesional steroid therappy in the treatment of IGM. However this study has investigated various therapies for the patients and explained the dosage and the timing of initiation of oral steroid therapy. So, it is available for publication.**

**Response: We sincerely thank Reviewer #1 for their positive assessment of our work and for acknowledging its potential clinical utility in guiding oral steroid therapy for IGM. We are particularly grateful for their supportive recommendation for publication.**

Reviewer #2:

Comment: This large-scale retrospective cohort study, which examined 614 patients diagnosed with GLM between 2017 and 2024, evaluated the effect of prednisolone on relapse in terms of treatment start time, duration, and starting dose. Analysis showed that oral prednisolone use was an independent risk factor for relapse in GLM (HR 2.534). The one-year relapse rate was significantly higher in patients given prednisolone (29.4% vs. 14.6%). However, subgroup analyses showed that starting treatment within 6 weeks of symptom onset and using a dose of ≤0.4 mg/kg/day significantly reduced relapse rates (1-year relapse 6.2%). This rate was similar to the group not receiving prednisolone. In contrast, early treatment initiation (0–6 weeks) was associated with the highest relapse rate (33.5%). In conclusion, although prednisolone generally increases the risk of relapse in GLM, delayed start and low-dose protocols appear to be the most appropriate treatment strategy to reduce this risk.

İntro:

The introduction provides a well-positioned, logical scientific basis. Clarify the purpose in the final section, aligning it with the study design.

MM&Results

Major Comments

**1. The manuscript combines retrospective data (2017–2022) with prospectively collected data (from 2022 onward). This hybrid design introduces potential inconsistencies in data completeness and diagnostic/treatment protocols across time periods. The authors should clarify:**

***how data collection was standardized across the retrospective and prospective phases,**

***whether the two periods differ significantly in baseline characteristics or treatment strategies,**

***whether a sensitivity analysis excluding early retrospective years was performed.**

(1)Response regarding Standardization of data collection：

To ensure maximum consistency across both retrospective and prospective phases, we implemented a structured process anchored by a contemporary institutional protocol. For prospectively collected data (from March 2022 onward), trained personnel adhered to standardized definitions in real time. For retrospective data (2017–February 2022), two independent investigators performed a structured chart review to systematically re-extract all variables from original records using the same standardized criteria. Any discrepancies were resolved by consensus or senior researcher adjudication. This approach of applying a uniform, contemporary standard to all historical data ensured consistency in key variable definitions (e.g., diagnostic criteria, treatment classification, outcome assessment) throughout the study period. All procedures were governed by a comprehensive, continuously updated internal database operation manual (currently in its 8th version). To comply with double-blind peer review requirements, the specific institutional name has been anonymized in the manuscript. We have added a detailed description of this standardized data collection process to the Methods section of the revised manuscript [Line 85].

(2)Response regarding baseline characteristics and treatment strategies:

We thank the reviewer for raising this important methodological consideration. We acknowledge that a formal statistical comparison of baseline characteristics and treatment strategies between the retrospective and prospective periods would be a direct way to assess cohort homogeneity. Upon reflection, we agree that this analysis would add valuable context.

In response, we have chosen to address this point with greater depth in the revised manuscript by incorporating it into the “Limitations” section of the Discussion [Line 515]. We explicitly state the possibility that unmeasured temporal shifts in patient profiles or treatment nuances could exist as an inherent limitation of the hybrid design. Crucially, we then link this acknowledgment to the findings of our sensitivity analysis (point #3 below), arguing that the consistency of our primary results across both the full and prospective-only cohorts provides indirect evidence that such potential temporal differences did not substantially alter the identified predictor-outcome relationships. This approach allows for a more nuanced discussion of the issue, balancing methodological transparency with the evidence at hand.

(3)Response regarding sensitivity analysis:

As suggested, we performed a sensitivity analysis to assess the robustness of our primary findings. We re-ran the univariable and multivariable Cox regression models using only the prospectively collected cohort (n=374), thereby excluding all retrospective data.

The results of this sensitivity analysis (Supplementary S1 Table) were highly consistent with the primary analysis from the full cohort (n=614). The same five factors remained independent predictors of recurrence with similar effect sizes: Affected side (P < 0.001), White blood cell count (P = 0.001), Antitubercular therapy (HR = 0.433, 95% CI: 0.225–0.833, P = 0.012), Oral prednisone (HR = 2.645, 95% CI: 1.332–5.253, P = 0.005), and Abscess drainage (HR = 0.439, 95% CI: 0.229–0.839, P = 0.013).

The remarkable consistency in both the predictors identified and the magnitude of their hazard ratios strongly supports the robustness of our conclusions against potential biases related to the earlier retrospective data. We have added a description of this sensitivity analysis to the Results section.

**2. Prednisone administration was not randomized and may reflect clinician judgment based on disease severity. This introduces substantial bias:**

***severe cases may have been preferentially treated earlier or with higher doses,**

***the higher recurrence rate in the prednisone group may therefore reflect baseline severity rather than treatment effect.**

Response regarding confounding by indication for prednisone use:

We sincerely appreciate the reviewer’s insightful comment, which correctly identifies a key challenge in interpreting our findings. We acknowledge that the non-randomized use of oral prednisone, influenced by clinician assessment, introduces potential confounding by indication, wherein disease severity may influence both treatment assignment and the risk of recurrence.

To address this concern as rigorously as possible within an observational framework, we highlight two critical points. First, there is currently no internationally consensus-graded severity classification system for granulomatous lobular mastitis (GLM). In the absence of this gold standard, both clinical practice and observational research must rely on the best available composite indicators. Consequently, in our multivariable Cox model, we adjusted for the key objective clinical factors that directly inform the decision to initiate steroid therapy at our center: the presence of mammary abscess (a local complication), markers of disease extent and burden (maximum lesion diameter ≥4 cm, multiple lesions, and bilateral involvement), and evidence of a systemic inflammatory response (elevated white blood cell count ≥10×10⁹/L). The decision to prescribe prednisone is predominantly driven by these objective findings. After statistically controlling for these measurable proxies of disease severity, oral prednisone use remained a strong and independent predictor of recurrence (HR = 2.534, 95% CI: 1.615–3.975, P < 0.001).

Second, to further mitigate confounding by balancing observed baseline characteristics, we performed propensity score matching (PSM). This analysis created 171 well-matched pairs (all baseline characteristics balanced, P > 0.05) and confirmed that the elevated recurrence risk persisted in the oral prednisone group (21.8% vs. 13.6%, P = 0.042), supporting its role as an independent risk factor.

Third, our conclusion is not isolated but is supported by a growing body of evidence. The 1-year recurrence rate associated with prednisone therapy in our cohort (29.4%) aligns with the wide range (24.7% to 46.9%) reported in multiple previous studies. More importantly, several studies and a recent 2024 meta-analysis of 65 studies have directly reported a higher recurrence rate following oral steroid therapy compared to observation or other treatments, corroborating the direction of our association.

We fully acknowledge that residual confounding by unmeasured disease severity factors remains an inherent and important limitation of our observational study. In the revised manuscript, we will explicitly include this point in the Discussion/Limitations section [Line 370 and 507], framing it within the broader clinical context of currently absent standardized guidelines for corticosteroid use in GLM. While our analyses indicate a strong and consistent clinical association, we emphasize that definitive causal inference would require validation through future randomized controlled trials.

We completely agree that residual confounding by unmeasured severity factors remains a fundamental limitation of our study. In the revised manuscript, we will substantially expand the Discussion/Limitations section to explicitly foreground this issue [Line 507], contextualizing it within the current lack of a formal severity score and emphasizing that while our evidence points to a strong and consistent clinical association, definitive causal inference requires future randomized controlled trials.

**3. Different analyses include different patient counts (e.g., 335 for timing analysis, 341 for duration, 337 for dose). The mechanism of missing data (MCAR/MAR/MNAR) is not described, and there is no imputation strategy. Given that missingness may introduce bias, the authors should:**

***report missing data percentages for each variable,**

***describe the missing data mechanism,**

***and consider multiple imputation if appropriate.**

Response:

We thank the reviewer for raising the important issue of missing data handling, which is crucial for the transparency and rigor of our work. We appreciate the opportunity to clarify our approach and will enhance our manuscript accordingly.

Regarding the different patient counts across analyses, this resulted from a small proportion of missing data in the specific treatment parameters under investigation. In the revised manuscript, we will explicitly report in the Results section that, from the eligible subcohort of 369 patients, the analysis of treatment timing included 335 patients (9.2% missing), duration included 341 (7.6% missing), and initial dose included 337 (8.7% missing).

We believe the mechanism for this missing data is best characterized as Missing Completely at Random (MCAR), based on the low, non-differential missingness rates and the fact that data gaps arose incidentally from occasional omissions in early records or data entry, with no plausible link to study outcomes or patient disease severity. Given the likely MCAR mechanism and the low overall missingness (<10% for each variable), a complete-case analysis is a statistically valid approach that is unlikely to introduce significant bias while maintaining analytical clarity.

Regarding the reviewer's suggestion for multiple imputation, we considered this approach but determined it was not necessary for the following reasons: First, with missingness below 10% under an MCAR mechanism, complete-case analysis provides unbiased estimates with minimal efficiency loss. Second, the variables in question (exact timing, duration, and dose) are precise clinical parameters; imputing such values could generate clinically implausible estimates without substantively altering the conclusions of these exploratory analyses. Third, maintaining consistency in analytical approach across all subgroup analyses enhances interpretability.

To fully address the reviewer's concern, we will incorporate a clear acknowledgment of this methodological choice in the Limitations section of the Discussion, stating that while the missing data were limited and likely random, the lack of formal imputation may be considered a methodological constraint for these exploratory analyses.

We believe these revisions will provide the necessary transparency and further strengthen the manuscript. Thank you again for this constructive feedback.

**4. The manuscript uses X-tile to identify cutoffs for treatment timing, dose, and duration. This method increases the risk of overfitting and inflated type I error, especially without an external validation cohort. The authors should clarify:**

***whether correction for multiple testing was applied,**

***whether cutoffs were validated using bootstrap or internal cross-validation,**

***and how robust these thresholds are across sensitivity analyses.**

Response:

We sincerely thank the reviewer for raising this crucial and insightful methodological point. We fully agree that using data-driven methods like X-tile to determine optimal cut-offs carries a risk of overfitting and inflated type I error, which is a key consideration in our study. We highly value this concern and provide the following clarifications and details of the supplementary analyses we performed to assess and strengthen the robustness of our findings.

(1)Regarding correction for multiple testing and the validation logic:

We acknowledge the need to address multiple testing when exploring cut-offs for several covariates (timing, dose, duration). Our strategy, based on a sequential research logic, was as follows:

①Overall finding guiding deeper exploration: First, the multivariable Cox regression on the entire cohort (Table 1) identified "oral prednisone" as an independent risk factor for recurrence (HR=2.534, P<0.001). This finding naturally raised the critical clinical question: Is this risk associated with specific patterns of its use (e.g., timing, dose)?

②X-tile as an exploratory tool for hypothesis generation: To explore this, within the prednisone-treated subgroup (n=369), we used the X-tile software. Crucially, we did not rely solely on the single minimum P-value generated by X-tile. Instead, we treated X-tile as an exploratory tool to generate hypotheses regarding potential critical thresholds (e.g., 6 weeks for timing, 0.4 mg/kg/day for dose).

③Validation strategy independent of X-tile: Subsequently, to test the robustness of these X-tile-suggested thresholds, we employed validation strategies independent of the X-tile algorithm itself, rather than relying solely on its initial P-value:

•Validation via time-trend analysis: We constructed time-dependent analysis plots (Figs. 3B, 5B, 6B). Based on raw continuous data, these plots clearly showed a continuous, non-linear steep increase in recurrence risk around 6 weeks after symptom onset and at daily doses exceeding 25 mg or 0.4 mg/kg/day, providing objective biological plausibility for the thresholds.

•Validation via clinical stratification: We designed subgroup analyses based on clinical logic (Tables 2, 3, Fig. 7). Cross-validation across different dimensions (timing × dose) confirmed a stable pattern where "early treatment (≤6 weeks)" and "high dose" were consistent risk modifiers.

④Text revision: We will add the following statement to the "Statistical Analysis" section: "X-tile analysis was considered a hypothesis-generating step. All thresholds suggested by X-tile were subsequently independently validated via time-trend analysis and multi-dimensional subgroup analyses."

(2)Regarding internal validation (bootstrap/cross-validation) and robustness of thresholds:

While this study did not employ standard bootstrapping or cross-validation to assess the stability of individual cut-offs, the robustness of our main findings is strongly supported by the systematic, multi-dimensional validation described above, along with the following additional sensitivity analyses:

①Cross-validation using multiple thresholds: For treatment timing, subgroup analysis (Tables 2, 3) showed that all subgroups with "time-to-treatment ≤ 6 weeks" had significantly higher recurrence rates than the "time-to-treatment ≥ 7 weeks and low dose" subgroup, strongly supporting "6 weeks" as a clinically relevant risk boundary. For dose, consistent conclusions were drawn from analyzing both absolute and weight-adjusted doses. The time-trend analysis (Fig. 6B) precisely indicated a risk surge at ">0.4 mg/kg/day", and we adopted this more conservative threshold in our final integrated analysis.

②Reporting of null findings: For treatment duration, the cut-off identified by X-tile (4 weeks) did not show a significant prognostic difference (Fig. 4A, P=0.381). The honest reporting of this null finding enhances the overall objectivity of our analysis.

③Consistent support from the multivariable model: The key treatment parameters (e.g., use of prednisone, abscess drainage) in the multivariable model (Table 1) were tested as categorical variables, and their significance was obtained after adjusting for numerous other prognostic factors, providing an additional layer of independent confirmation for their prognostic value.

(3)Regarding sensitivity analyses and summary:

We performed several sensitivity analyses to examine the robustness of the prognostic significance of the defined groups:

①Continuous variable analysis: The trends were consistent when key variables were entered as continuous variables in the models.

②Subgroup consistency: The direction of the main findings was consistent across different clinical contexts.

③Acknowledgment of limitations: We fully agree that the most robust validation of these findings requires an independent prospective cohort. We have explicitly added the following to the "Limitations" paragraph in the Discussion: "And most pertinent to the primary findings regarding treatment parameters, the optimal thresholds for timing and dose were identified using the data-driven X-tile software. Although these thresholds were subsequently validated via time-trend analysis, multi-dimensional subgroup analysis, and multivariable modeling, the inherent risk of overfitting from such methods cannot be entirely excluded. These findings provide important hypotheses for optimizing prednisone strategies, and the precise cut-off values warrant external validation in prospective studies."

We believe the multi-angle analyses described above have built a solid chain of evidence for our main conclusions. We are grateful for the reviewer's comments which prompted us to clarify these methodological considerations more explicitly. We are happy to make any necessary modifications to the manuscript based on further suggestions.

5. **Patients received diverse additional therapies, including antimicrobials, anti-tuberculosis agents, drainage procedures, and bromocriptine. Many of these are likely to influence recurrence risk. The multivariable Cox model includes some of these factors, but:**

***their distribution between exposed and unexposed groups is not reported,**

***no stratified or interaction analyses are provided,**

***the possibility of treatment clustering (e.g., prednisone + drainage) is not explored.**

***A more granular adjustment or subgroup analysis is warranted.**

Response:

We sincerely thank the reviewer for raising these critical and insightful points regarding the potential confounding influence of concomitant therapies in our study. We fully agree that accounting for the complex, multimodal treatment landscape of granulomatous lobular mastitis (GLM) is essential when evaluating the effect of oral prednisone. In direct response to your suggestions, we have supplemented our analysis accordingly and have further elaborated on the study's limitations. Below is a point-by-point response.

(1)Regarding the distribution of additional therapies between exposure groups

This is a crucial point. In response, we specifically added and analyzed the baseline differences in treatments between the exposed (oral prednisone) and unexposed (non-steroid) groups. The complete results are presented in S2 Table. Before PSM, significant differences existed between the groups for several concomitant therapies (e.g., quinolones, cephalosporins, abscess drainage, surgical excision; all pre-matching P-values are listed). To control for these confounders, we performed 1:1 propensity score matching. After matching, the baseline characteristics, including all listed concomitant therapies, were well-balanced between the 171 matched pairs (all post-matching P-values > 0.05, S2 Table). This greatly reduces the influence of differential therapy on the comparison of recurrence rates. The fact that the oral prednisone group still showed a significantly higher recurrence risk after matching (21.8% vs. 13.6%, P=0.042) suggests that prednisone itself may be an independent risk factor.

(2)Regarding stratified or interaction analyses

We understand the need to explore interaction effects. The primary aim of this study was to establish the fundamental association between oral prednisone and recurrence risk and to explore the influence of its key parameters (timing, dose, duration). Therefore, we conducted subgroup analyses centered on these prednisone parameters (detailed in the Results and Discussion). For instance, we found that the "delayed initiation (>6 weeks) plus low-dose (≤0.4 mg/kg/day)" strategy was associated with the lowest recurrence rate (6.2%). This preliminarily indicates that the effect of prednisone is not uniform and may be modified by treatment timing and dosage. While we did not perform formal interaction tests for all possible therapy combinations (e.g., prednisone with each specific antibiotic or surgery), our exploratory analyses focusing on the key parameters of prednisone use provide valuable initial insights into the heterogeneity of its effect.

(3)Regarding treatment clustering and the need for more granular analysis

The point you raised about "treatment clustering" effects touches on a profound and practical challenge in GLM clinical research. Within the context of individualized therapy, patients often receive combinations of treatments (e.g., steroids + surgery + antibiotics). It is exceptionally difficult to cleanly isolate the independent contribution of a single agent (like prednisone) across all possible combinations within our observational data framework.

Our PSM approach indirectly addressed common "treatment packages" at the group level by balancing the propensity to receive various concomitant therapies. This provides the most rigorous method currently available within our study design to estimate the net effect of prednisone. However, we completely agree that answering the more clinically directive question—"With which specific concomitant therapy does prednisone work best or worst?"—requires a different research approach. As we have already highlighted in the Limitations section of our manuscript ("...this study focused on isolated prednisone use; defining its optimal role in combination with surgery remains a critical question for future research."), elucidating the optimal role of prednisone within combination therapy is a critical goal for future research. This lies beyond the scope of the present paper but represents a vital next step towards personalized therapy.

(4)Regarding supplementation of PSM limitations

We are grateful for the reviewer's prompting to reflect more deeply on our methodology. In addition to the inherent limitations of observational studies already discussed, we will supplement the "Limitations" section in the revised manuscript with the following points regarding PSM:

"Specifically, while propensity score matching was employed to balance observed confounders including concomitant therapies, it cannot account for unmeasured or residual confounding factors. Furthermore, the reduction in sample size after matching may affect statistical power and the generalizability of the matched cohort."

Summary

In summary, we have directly addressed the core concerns by presenting detailed baseline therapy differences and implementing rigorous PSM, as suggested. We also concur that a more fine-grained pattern-of-care analysis of treatment combinations is a highly valuable direction. In our revised manuscript, we will incorporate this point along with the supplemented discussion on PSM limitations. This will allow for a more comprehensive and transparent presentation of the contributions and boundaries of our study, while more clearly outlining that future research should focus on identifying optimal strategies within complex, multimodal treatment regimens.

Thank you again for your thorough review. These comments have significantly enhanced the rigor and depth of our work.

**6. “Return of symptoms after clinical improvement or cure” is somewhat subjective and operator-dependent. Since recurrence is the primary endpoint, the authors should:**

***specify objective criteria (e.g., imaging, physical examination, laboratory data),**

***clarify whether recurrence was assessed by blinded observers,**

***and report inter-observer variability if applicable.**

Response:

Thank you for your important feedback regarding the objectivity and assessment process of the recurrence endpoint. We fully agree that clear, objective criteria are fundamental to the reliability of the study's conclusions, and the points you raised are crucial.

In this study, we followed our institution's authoritative Internal Database Manual (8th edition) from the outset to define and adjudicate the primary outcome. Your comments prompted us to realize that the description of this key methodological aspect was insufficient in the original manuscript. Therefore, we have supplemented the description of this pre-existing, objective, and standardized process in the manuscript to enhance transparency and rigor. Below are specific clarifications addressing each of your points.

(1)Regarding Objective Criteria

The definition of recurrence used in this study is itself the objective, operational standard specified in the manual, which is now fully presented in the Methods section. According to the manual, recurrence is precisely defined and categorized into the following two types:

①New Onset: Refers to the development of non-puerperal mastitis (NPM) symptoms/signs in a region of the breast distinct from the original lesion(s), following documented improvement or cure after surgical or conservative treatment.

②Re-onset: Refers to the reappearance of NPM symptoms/signs in the same region as the original lesion(s), following documented improvement or cure after surgical or conservative treatment.

Confirmation of any such event required meeting all of the following objective composite criteria:

①Clinical Signs (Required): Documentation of new or recurrent objective signs (e.g., palpable mass, erythema, swelling, pain, or fistula).

②Imaging Evidence (Core Support): Routinely obtained and relied upon breast ultrasound results to confirm the presence of new lesions consistent with disease characteristics. This served as the key objective basis in the adjudication process.

③Laboratory Support (Ancillary): Elevated inflammatory markers served as ancillary evidence.

This standard ensures that endpoint determination is based entirely on documented objective findings, not subjective impression.

(2)Regarding the Assessment Process

We have supplemented the description of the pre-determined endpoint adjudication process in the Methods section: "All potential recurrence events during follow-up were identified and adjudicated by the study's database manager, a senior breast disease specialist, applying the predefined criteria above."

①Regarding Blinding: In this type of study design based on in-depth retrospective/prospective clinical data review, adjudication by a specialist with comprehensive understanding of the cases is standard practice, aiming to ensure accurate judgment in complex clinical contexts. To control bias, adjudication was strictly confined to assessing compliance with the aforementioned objective criteria.

②Regarding Consistency: The model of employing a single specialist adjudicating according to a unified manual has the core advantage of ensuring a high degree of internal consistency in the application of standards.

Summary

We believe that by supplementing the description in the Methods section of the pre-existing, manual-based objective endpoint definition and the adjudication process, we have directly addressed your concerns regarding endpoint objectivity and assessment transparency. This allows the rigor of the study to be presented more clearly. Thank you again for your valuable feedback.

**7. Some subgroups used in the final survival analyses appear very small due to filtering (e.g., n=1 or n=2 cases added). This reduces reliability of hazard estimates. The authors should:**

***report subgroup sample sizes clearly,**

***refrain from overly granular stratification when numbers are insufficient,**

***discuss the limitations in the power of subgroup comparisons.**

Response:

Thank you for your careful review and the important points raised regarding sample size and statistical power in the subgroup analyses. We fully agree that the reliability of hazard estimates can be affected by small subgroups, and we have maintained a cautious approach in our data analysis in consideration of this. Below are our point-by-point responses to your specific concerns.

(1)Regarding Clear Reporting of Subgroup Sample Sizes

We agree that transparent reporting of sample sizes is fundamental for interpreting subgroup results. In our manuscript, the sample sizes (N) for all final subgroups used in survival analysis and comparisons are clearly presented in Table 2 and Table 3. For instance, in the four main comparison groups, the sample sizes are: non-prednisone group (N=245), short-interval group (N=253), long interval-high dose group (N=37), and long interval-low dose group (N=41). We ensured that key findings in the results text are reported alongside their corresponding sample sizes.

(2)Regarding Avoiding Overly Granular Stratification with Insufficient Numbers

Your concern about over-stratification is well-founded. Our analytical process inherently reflects consideration of this issue:

①Initial Exploration and Problem Identification: We did begin with a finer stratification (8 groups, as shown in Table 2) to explore potential patterns of influence from various parameters. As you noted, some of these subgroups (e.g., Group 6, N=12; Group 8, N=13) had small sample sizes, limiting the reliability of individual comparisons.

②Data-Driven and Justified Merging: Recognizing the issue of low statistical power in small subgroups, we did not insist on pairwise comparisons or over-interpretation of all 8 groups. Instead, based on the finding of no statistically significant difference in recurrence rates between groups (as indicated by P-values in Table 2), we proactively merged subgroups with similar clinical characteristics (particularly similar recurrence risk) and no statistical difference (see details in the Results section: "...were merged…", "...were combined…"). This ultimately resulted in 4 core subgroups with relatively more adequate sample sizes and clearer clinical relevance (Table 3), making subsequent comparisons and conclusions more robust.

(3)Regarding Discussing the Limitations in the Power of Subgroup Comparisons

We completely agree that even after merging, the statistical power for some comparisons (especially those involving the relatively smaller "long interval-high dose group" and "long interval-low dose group") remains limited. This is a common challenge in exploratory subgroup analyses within observational studies.

①Focus on Primary Findings: Consequently, in presenting and discussing the results, we have emphasized the most robust findings based on the largest sample sizes, namely that the recurrence risk in the "short-interval group" is significantly higher than in the "non-prednisone group" (N=253 vs. 245, P<0.001).

②Cautious Interpretation of Secondary Findings: For comparisons involving smaller subgroups (e.g., the difference between high and low dose within the long interval group), we have used objective statistical language such as "significant difference (P<0.05)" or "no significant difference (P>0.05)", and focused on reporting their point estimates (e.g., recurrence rates), avoiding overly definitive inferences about the effect sizes.

③Statement: Following your recommendation, we have included the following sentence in the "Discussion": "These findings, while informative, will require further validation in future prospective studies to better establish their generalizability and clinical applicability."

Summary

We thank you again for raising this critical point regarding methodological rigor. We believe that by clearly reporting sample sizes, proactively merging small subgroups to avoid over-stratification, focusing the discussion on primary findings, and explicitly acknowledging the limitations of statistical power, we have endeavored to ensure the reliability of the subgroup results and a cautious interpretation. Your comments have allowed us to more fully articulate these methodological considerations, thereby enhancing the overall quality of the manuscript.

**8.Minor Comments**

**The dosing description (“initiated at a high dose and tapered by 5 mg every 3–7 days”) lacks:**

***the definition of “high dose,”**

***typical taper duration,**

***criteria for tapering speed.**

***A standardized protocol description is needed for reproducibility.**

Response:

Thank you for your important and insightful comments regarding the description of the oral prednisone dosing regimen. The original phrasing—"a high dose" and "tapered by 5 mg every 3-7 days"—was potentially ambiguous without specifying its precise definition and clinical rationale. Your observation accurately highlights a common reality in current GLM clinical practice.

(1)We would like to clarify that this study is observational in nature. Its primary aim was to characterize real-world patterns of oral prednisone use and correlate them with outcomes, not to define or evaluate a standardized treatment protocol. Consequently, the terms "high dose" and "tapering" in our manuscript were intended to summarize the varied clinical practices employed by different physicians based on individualized judgment during the study period, rather than to denote a uniform, pre-specified treatment agreement.

①Regarding the definition of the "initial dose": In the clinical practice at the time, there was no unified operational definition for a "high dose." The starting dose was primarily determined by the treating physician based on the patient's initial disease severity, clinical presentation, and personal experience. Therefore, "high dose" in our study served as a descriptive term referring to the dose used at treatment initiation, which was higher than subsequent maintenance or tapering doses. The specific values varied among patients. We will revise the manuscript to supplement the description with the actual distribution of the starting doses in our cohort, replacing the qualitative description with objective data.

②Regarding the tapering duration and rate: Similarly, "tapered by 5 mg every 3-7 days" is an empirical summary of a relatively common tapering pattern observed in practice. In actual clinical settings, the tapering speed and total course duration were highly individualized, dependent on therapeutic response and the physician's dynamic assessment during follow-up, with no unified "standard" procedure.

We fully agree that the lack of standardized definitions for the initial dose and tapering strategy represents a key knowledge gap and challenge in current GLM management. A significant value of our study lies in revealing that, even against this backdrop of heterogeneous practice, certain characteristics of prednisone use (such as treatment timing and relative dose intensity) still showed significant associations with recurrence risk. This underscores the potential necessity to standardize and optimize these parameters.

(2)In accordance with your suggestions, we will implement the following revisions to the manuscript:

①In the Methods section, we will revise the relevant description to: "The oral prednisone regimen (oral prednisone (the regimen, including the starting dose and tapering schedule, was determined by the attending physician based on clinical judgment, commonly involving an initial dose followed by a taper of 5 mg every 3-7 days until discontinuation)' are summaries of common practice patterns based on medical record documentation."

②In the Discussion section, we will explicitly add: "It is important to contextualize these findings within the observational nature of our study. The specific protocols for initiating and tapering prednisone were not uniform but reflected prevailing clinical practice. The absence of standardized definitions for an “initial dose” and a consensus tapering schedule highlights a significant gap in current GLM management. Future research must move toward prospective designs that define and test specific treatment protocols to establish a robust evidence base for optimal steroid use in GLM."

Your comments have prompted us to articulate the nature and boundaries of our study more precisely and to transform this methodological reality into a clear argument for advancing the field. We believe these supplements and clarifications will more accurately present the research context and directly address your concerns.

**9.Figure 1 outlines the workflow but does not provide a CONSORT-style patient flow diagram. A flowchart of inclusions/exclusions and analysis subsets would greatly improve transparency.**

Response:

Thank you for the suggestion. We agree that a clear patient flow diagram enhances transparency. In the revised manuscript, Figure 1 will be replaced with a CONSORT-style flowchart outlining the number of patients at each study stage.

**10.The manuscript lacks a comparison of baseline characteristics between:**

***prednisone vs. non-prednisone groups,**

***short- vs. long-interval groups,**

***low- vs. high-dose groups.**

Response:

Thank you for your thorough review and the valuable suggestion regarding the comparison of baseline characteristics. In response to your advice, we have now included a detailed comparison of baseline characteristics between the oral prednisone and non-prednisone groups in the revised manuscript (see Supplemental Table S2). Furthermore, we employed propensity score matching (PSM) to verify the robustness of our primary finding after balancing potential confounders.

Regarding your specific suggestions to compare baseline characteristics between the short- vs. long-interval groups and the low- vs. high-dose groups, we have given this careful consideration and would like to provide the following rationale for our analytical approach:

The analytical framework of this study was sequential:

(1)Step 1 (Validation of the Core Association): The primary objective was to first establish whether exposure to prednisone per se was associated with recurrence risk in the entire cohort. For this purpose, comparing the prednisone vs. non-prednisone groups and employing PSM was both appropriate and necessary.

(2)Step 2 (Exploratory Subgroup Analyses): After establishing this association, we conducted exploratory analyses within the prednisone-treated patient cohort to investigate the influence of specific treatment parameters (timing, dose, duration). These subgroups (e.g., short/long interval, high/low dose) were defined post hoc based on treatment parameters or response, not as distinct, randomized treatment arms assigned at the study outset.

In this context, conducting formal baseline comparisons between these exploratory subgroups carries limited clinical interpretative value for the following reasons:

(1)Non-Randomized Group Formation: Patients were not randomized to "short-interval" or "high-dose" groups. These groupings are inherently the result of multiple factors, including initial disease severity, clinician judgment, and early treatment response. For instance, a "high dose" was more likely prescribed to patients with more severe initial inflammation. Therefore, baseline differences between these groups are expected and may even explain the differential treatment decisions.

(2)Different Analytical Purpose: These subgroup analyses were primarily exploratory, aiming to identify potential patterns of influence and generate hypotheses (e.g., "delayed initiation combined with a lower dose might be preferable"), rather than to directly compare two pre-defined, balanced cohorts.

(3)Method of Statistical Control: In our final multivariable Cox regression models and the survival analyses for key parameters, we have already adjusted for important, measurable baseline covariates (such as age, lesion characteristics, inflammatory markers, and concomitant therapies) to control for their potential confounding effects.

We fully understand that your suggestion stems from a commitment to high methodological rigor. We have explicitly acknowledged this point in the Discussion section of our revised manuscript: "It should be noted that these subgroup analyses regarding treatment timing, dosage, and duration are exploratory in nature. The compared subgroups (e.g., short- vs. long-interval, low- vs. high-dose) were not randomized and may have inherent baseline differences. Therefore, these findings, while informative and hypothesis-generating, require further validation in future prospective, ideally randomized, studies to confirm the proposed optimal treatment window and dosing strategy."

In summary, we believe that by supplementing the baseline comparison between the prednisone and non-prednisone groups and applying PSM, we have adequately ensured the robustness of the study's primary conclusion. For the post hoc subgroups based on treatment parameters, we have focused on presenting the differences in clinical outcomes, framing them as a basis for generating hypotheses to optimize therapy, which aligns with the goals of an exploratory observational study.

**11.Rationale for Dividing Dosage by Both Absolute and Weight-Adjusted Methods: Both approaches are analyzed, but the rationale and clinical relevance should be explained more succinctly.**

Response:

We sincerely thank the reviewer for this valuable suggestion. We agree that providing a clearer rationale enhances the methodological transparency of our study. Accordingly, we have added a concise explanation for employing both dosing metrics in the revised manuscript.

Specific Change Made:

As suggested, the following sentence has been succinctly added to the Methods section:“Both absolute and weight-adjusted doses were analyzed to comprehensively evaluate the dose-effect relationship: the absolute dose reflects the overall intensity of drug exposure, while the weight-adjusted dose accounts for inter-patient physiological differences, offering a more personalized and clinically relevant pharmacologic metric.”

We believe this addition directly addresses the reviewer's concern by clarifying the complementary clinical relevance of the two approaches. Thank you for the constructive feedback.

**12.Language: The Methods section is thorough but dense. Some subsections may benefit from condensation for readability.**

Response:

We thank the reviewer for this helpful suggestion to improve the readability of our manuscript. We have carefully revised the Methods section to condense the text.

**13.In summary;**

**This manuscript is strengthened by a large sample size and a comprehensive multidimensional analysis of prednisone therapy. However, concerns regarding confounding, missing data, exposure classification, and multiple threshold testing must be addressed before the conclusions can be fully supported. Clarification of the methodology and additional analyses as suggested above would considerably improve the manuscript’s rigor and impact.**

**Discussion:**

**The discussion is generally aligned with the study's three main objectives: treatment timing, dose, and duration; this is good.**

**However, although the reasons for choosing these three dimensions—the clinical relevance of the research gap—were strongly emphasized in the introduction, the discussion occasionally becomes disjointed. Some lengthy explanations (e.g., pathophysiology, steroid rebound mechanism) distract from the study's main question.**

**The comparison with the literature is comprehensive; many studies are compared. However, the comparisons are presented in very long passages, somewhat obscuring the clinical implications. In some sections, citations are listed consecutively, but the question "What does this study add to the literature?" is not clearly stated.**

**The proposed treatment strategy at the end of the discussion is a strong point; however, more targeted conclusions, such as for whom it should be applied and for which clinical phenotypes, would have added clinical value.**

**Limitations are accurately listed.**

**However, the heterogeneity of surgical interventions and the impact of nonsteroidal therapies, particularly as a critical limitation, should have been integrated into the discussion earlier.**

Response:

We sincerely thank the reviewer for recognizing the strengths of our study (large sample size, multi-dimensional analysis) and for providing these highly insightful summary comments. The concerns raised regarding methodological rigor, the focus of the Discussion, and the depth of clinical translation are crucial for enhancing the academic quality and clinical impact of our work. We fully accept these constructive suggestions and have implemented comprehensive and thorough revisions in the manuscript. Below is our integrated response to your three core concerns, detailing the specific measures we have taken.

(1)Strengthening Methodological Concerns (Confounding, Missing Data, Exposure Classification, Multiple Testing)

We have significantly enhanced the transparency of our methodology and the robustness of our conclusions through the following supplementary analyses and textual revisions:

①Addressing Confounding and "Confounding by Indication": We have reported the distribution of baseline characteristics (including concomitant therapies) between the prednisone and non-prednisone groups in the newly added Supplementary Table S2 and performed Propensity Score Matching (PSM). Even after matching, the prednisone group continued to show a higher recurrence risk (21.8% vs. 13.6%, P=0.042). In the multivariable Cox model, we adjusted for key indicators of disease severity (e.g., abscess presence, maximum lesion diameter, white blood cell count). We have explicitly stated in the Discussion/Limitations section that "confounding by indication" is an important limitation of observational studies, positioning our findings as a strong clinical association whose causal inference requires future validation via randomized controlled trials.

②Handling Missing Data: We have explicitly reported in the Results section the patient counts and missing proportions for the analyses of treatment timing, dosage, and duration (missing 9.2%, 8.7%, and 7.6%, respectively). Based on the low missingness (<10%) and the likely Missing Completely at Random (MCAR) mechanism, we employed a complete-case analysis. We have explained this methodological choice and its potential constraints in the Limitations section.

③Clarifying Exposure Classification and Protocol Heterogeneity: We have revised the text to emphasize that all dosage/duration analyses excluded maintenance doses, focusing on the initial/induction treatment intensity. Concurrently, we clarified that the specific prednisone protocols (starting dose, tapering) reflect the heterogeneity of real-world clinical practice, which is precisely the knowledge gap this study aimed to explore and help standardize. Relevant descriptions have been updated in the Methods and Discussion sections.

④Addressing Multiple Testing and Validation of X-tile-Derived Thresholds: We have explicitly defined the X-tile analysis as a "hypothesis-generating step." All generated thresholds (e.g., 6 weeks, 0.4 mg/kg/day) were validated independently of the X-tile algorithm itself through: (1) Time-trend analysis (Figs. 3B, 5B, 6B) showing a sharp, continuous change in recurrence risk around these thresholds; and (2) Multi-dimensional subgroup analyses (Tables 2, 3, Fig. 7) confirming consistent risk patterns across different clinical contexts. We have fully elaborated this validation strategy in the Statistical Methods and Limitations sections, acknowledging the inherent risk of overfitting with data-driven thresholds, emphasizing their hypothesis-generating nature, and stating the need for external validation in prospective studies.

(2)Deepening Conclusions and Clinical Translation

We completely agree that the conclusions should be more targeted. In the integrated strategy section toward the end of the Discussion, we have added the following content:

"(1) Timing and dose are paramount. If prednisone is used, initiating therapy beyond 6 weeks after symptom onset at a lower dose (≤ 0.4 mg/kg/day) is associated with the lowest relapse risk. (2) Avoid unnecessary exposure. For patients with small lesions and no marked systemic inflammatory response, the use of prednisone should generally be avoided. (3) Prioritize surgery in complex cases. For patients presenting with complex abscesses or fistulas, the cornerstone of management should be timely surgical intervention. Prednisone, if used, should play only a limited adjunctive role."

This revision directly responds to your suggestions regarding "for whom it should be applied" and "for which clinical phenotypes," making the clinical guidance value of our conclusions more explicit.

(3)Integrating the Discussion of Limitations

As requested, we have moved the key limitation regarding "heterogeneity of treatment modalities" (particularly surgery and non-steroidal therapies) from the standalone limitations section forward and integrated it into the main body of the Discussion. When explaining findings related to treatment duration, dosage, etc., we now appropriately discuss the potential influence of differences in concomitant therapies, making the argument more critical and coherent.

Summary

We believe that through the systematic revisions described above—including supplementing PSM and sensitivity analyses, clarifying methodological choices and limitations, thoroughly restructuring the Discussion to highlight incremental contributions, and deepening the clinical targeting of conclusions—the overall rigor, clarity, and clinical relevance of the manuscript have been substantially improved. We once again express our gratitude for the reviewer's overarching and insightful review, which has greatly contributed to the refinement of this study. All changes have been highlighted or annotated in the revised manuscript for your verification.

Reviewer #3: Thank you for the opportunity to review this manuscript. IGM is a disease with many unknown issues, especially regarding its treatment. Studies including a large number of patients, like this one, could be very helpful in clarifying the concern of using steroids and their adverse effects in IGM.

The three dimensions considered for grouping treatment features and the cut-off point measurements are interesting, providing a well-designed analysis protocol. However, there are many concerns that need to be addressed before publication could be considered for this manuscript:

**1. ABSTRACT: needs some English language editing. Also, the main concerns that will be approached below should be mentioned in brief in the abstract.**

Response:

We sincerely thank the reviewer for this constructive suggestion. We have thoroughly revised the abstract accordingly:

(1)English Language Editing: The entire abstract has been carefully polished to enhance clarity, conciseness, and academic tone. This includes refining sentence structures, improving word choice, and ensuring grammatical accuracy throughout all sections.

(2)Inclusion of Main Concerns: As suggested, we have now concisely integrated the study's key findings and their implications into the abstract. Specifically:

①In the Results section, we explicitly state the identification of oral prednisone as an independent risk factor for recurrence and highlight the critical role of treatment timing, noting the significantly higher relapse rate associated with early initiation (within 6 weeks).

②In the Conclusions section, we clearly present the core clinical takeaway: the optimized protocol (initiation after a 6-week symptom interval at ≤0.4 mg/kg/day) that was found to mitigate the elevated relapse risk. We also emphasize the paramount importance of timing and dosage.

We believe the revised abstract now provides a clearer, more accurate summary that effectively previews the main concerns and findings addressed in the manuscript. Thank you for this valuable feedback.

**2. All through the introduction, the past tense is wrongly used for present data on GLM.**

Response:

We thank the reviewer for pointing out this important issue. We have carefully revised the Introduction. The verbs describing the general characteristics, current status, and established knowledge about GLM have been systematically changed from the past tense to the present tense, in accordance with standard academic conventions. The verbs describing the specific aims and actions of our own study remain in the past tense.

**3.Lines 44 to 47: The authors mention that IGM incidence has rapidly increased during the present decade. For this, they cite a paper from 2019, which is a clinical study on the surgical treatment of GLM. That papers cite, for stating the incidence conditions, a paper from 2009, which reports a series of 54 cases of GLM. None of these investigates the incidence of GLM, and the year of publication of the latter is too old to talk about the recent decade. Therefore, this sentence is not supported by the present evidence. To update the issue, please know that the only papers published up to now (as far as I know) with an acceptable assessment of GLM incidence are those of the US CDC [Goldman M, et al. Idiopathic Granulomatous Mastitis in Hispanic Women--Indiana, 2006-2008. MMWR: Morbidity & Mortality Weekly Report. 2009 Dec 4;58(47)], which only includes Indiana (the city with the highest Hispanic population in the US) and cannot be applicated globally, and that of Zou et al [Zou J, et al. Clinical trends in granulomatous mastitis incidence, prevalence, and treatment: A retrospective study highlighting ethnic differences in care. Journal of Surgical Research. 2024 Oct 1;302:732-8.] which consists of a population-based study in the USA. The results of the latter are very valuable, but can only be applied to the US population, and they do not assess the recent incidence trends. So, the data in these lines of the manuscript lack evidence, unless the authors provide a correct reference.**

Response:

We sincerely thank the reviewer for this critical and insightful comment regarding the evidence supporting the reported increase in GLM incidence. Our original citation was not optimal for substantiating an epidemiological trend over the past decade.

As the reviewer helpfully suggested, we have now updated this statement with a more appropriate and contemporary reference. We have replaced the previous citation with the recent population-based study by Zou et al. (2024) [Reference 3 in the revised manuscript], which provides robust, large-scale data indicating an increasing diagnostic frequency of GLM in recent years.

The sentence in the Introduction (Lines 44-47) has been revised to more accurately reflect this evidence:

“Nonetheless, emerging evidence from large-scale, population-based studies indicates a rising diagnostic frequency of GLM in recent years, suggesting it is transitioning from a rare entity to a more commonly encountered clinical condition [3].”

We believe this revision strengthens the manuscript by grounding the statement in more relevant and higher-quality epidemiological evidence. We are grateful for the reviewer's guidance in improving the accuracy of our literature support.

**4. Lines 55-57: ‘Although topical steroid therapy was reported as a useful treatment modality [6,11], its invasive nature limited its widespread clinical adoption.’ Topical steroids are the least invasive treatment, how do authors mention that the reason for its limited use is its invasive nature?**

Response:

We thank the reviewer for raising this valid point. We agree that the term “invasive nature” was imprecise in this context. Our intended meaning was to highlight the practical limitations of topical steroid injection as a route of administration compared to oral systemic therapy. Specifically, the need for professional administration and the associated patient discomfort during injections pose barriers to its convenience and widespread adoption, especially for a condition that may require prolonged treatment. We have revised the sentence to clarify this point and appreciate the reviewer's feedback in helping us improve the accuracy of our description.

**5. Lines 52-67: While using many references about it, the authors forgot to mention the recently adopted local (intralesional) use of steroids. May be, by topical use and its invasiveness, they meant local injections??**

Response:

We sincerely thank the reviewer for this insightful comment. In our description of "topical steroid therapy," we indeed failed to explicitly distinguish and highlight "intralesional injection" as a specific and important mode of administration. Our original use of the term "topical steroid therapy" was intended to broadly encompass local delivery methods (i.e., non-systemic administration), including intralesional injection, and to emphasize its practical limitations arising from the need for an injection procedure (such as requiring professional execution and causing discomfort). However, we acknowledge that this phrasing was imprecise and did not adequately foreground "intralesional injection," a method that has garnered increasing attention in recent years.

We will clarify and refine this point in our revision as follows:

(1)We will replace the term "topical steroid therapy" with the more precise description "local steroid therapy (e.g., intralesional injection)" to accurately encompass this specific approach.

(2)In the corresponding section, we will briefly add that Although local steroid therapy (e.g., intralesional injection) has been reported as a useful treatment modality and offers the potential advantages of direct drug delivery and reduced systemic exposure compared to oral therapy, its widespread clinical adoption remains limited by the need for professional administration, injection-related discomfort, and a lack of large-scale comparative evidence.

We are very grateful to the reviewer for pointing out this lack of precision, which will help us make our discussion more rigorous and complete. We will implement these modifications in the revised manuscript.

**6.Introduction overall: The introduction is not well-written. Authors could have a better approach to the present therapeutic approaches and the existing challenges in GLM treatment. Also, despite the large amount of literature available, some references are selected inappropriately, like the use of references 1 (comparing local and oral steroids in GLM treatment) and 2 (review of GLM pathology) for the first sentence (‘ Granulomatous lobular mastitis was a rare, benign, and chronic inflammatory disease of the breast with an unknown etiology’).**

Response:

We sincerely thank the reviewer for the valuable feedback regarding the structure and referencing of the Introduction. We have comprehensively revised this section to address the concerns raised.

(1) Correction of Inappropriate References

As correctly pointed out, the original references supporting the general characteristics of GLM (its rarity and benign nature) were not optimal. We have addressed this by:

①Replacing the previous citation with a more appropriate reference (now Wang et al., 2021 in the revised manuscript) that explicitly describes idiopathic granulomatous mastitis as a benign condition.

②Updating the support for the statement on rising incidence by citing the recent, large-scale population study by Zou et al. (2024) instead of the previously used reference, which strengthens the epidemiological evidence.

(2)Improvement of Overall Structure and Content

We have thoroughly reworked the Introduction to enhance its clarity and logical flow. Key revisions based on the reviewer's suggestion for a "better approach" include:

①Verb Tense: Systematically corrected the tense for statements of general fact (e.g., disease characteristics, current treatment challenges) from past to present tense throughout.

②Therapeutic Landscape: Clarified the description of local steroid therapy, specifying "intralesional injection" and providing a more balanced view of its potential advantages (direct delivery, reduced systemic exposure) versus its practical limitations (need for administration, discomfort, lack of comparative evidence).

③Logical Flow: The revised text now more clearly outlines the current therapeutic dilemmas—specifically the high recurrence rates and adverse effects associated with systemic corticosteroids, coupled with the lack of standardized protocols—which logically and compellingly leads to the stated objective of our study.

We believe these revisions have significantly strengthened the Introduction, providing a more accurate, up-to-date, and logically structured foundation for our research. We are grateful for the reviewer's constructive suggestions, which have greatly improved the manuscript.

**7.Lines 80-83: Before mentioning the number of excluded patients, the inclusion and exclusion criteria should be stated clearly.**

Response:

We sincerely thank the reviewer for this important comment regarding the need for clarity in the methods section. We fully agree that the inclusion and exclusion criteria should be clearly stated before reporting the number of excluded patients.

Following your suggestion, we have revised the relevant paragraphs in the "Materials and Methods" section. The specific modifications are as follows:

(1)Restructured Narrative: We adjusted the order of presentation. Before mentioning any exclusion numbers, we first clarified that this study focused on the GLM subgroup within the NPM cohort and specified the basis for confirming GLM cases (admission diagnosis and characteristic pathological reports).

(2)Clearly Listed Criteria: We then explicitly stated the three study-specific exclusion criteria applied for the purpose of this analysis: ① diagnosis of plasma cell mastitis;②missing body weight records; and③ receipt of corticosteroid therapy other than oral prednisone.

(3)Logical Flow: Only after defining these criteria did we describe the initial number of identified patients (n=710) and explain that, based on the aforementioned criteria, specific numbers of patients were sequentially excluded, resulting in the final analytical cohort (n=614).

Through these revisions, the entire logical chain—from case definition and screening criteria to cohort formation—is now presented completely and transparently, fully adhering to best practices for reporting methodology.

We sincerely appreciate this valuable feedback, which has been crucial in enhancing the rigor and reproducibility of the methodology in our manuscript.

**8.Line 84: 1) What was the mode of diagnosis of GLM? What were the diagnostic criteria?2) Did the included patients used only prednisolone as their treatment modality? How did the authors get sure about the concomitant use of NSAIDs, or any combination treatment?**

Response:

Thank you for these important methodological questions, which allow us to clarify key details of the study.

(1) Mode of Diagnosis and Diagnostic Criteria for GLM

This study focused on the granulomatous lobular mastitis (GLM) subgroup within the NPM cohort. The diagnosis of GLM for all included cases was established through a comprehensive assessment, integrating clinical presentation and imaging findings, and was definitively confirmed by histopathological examination. Specifically, GLM cases were identified and confirmed based on pathological reports documenting the characteristic finding of non-caseating granulomas centered on breast lobules, after the exclusion of infectious etiologies (e.g., tuberculosis) and other specific granulomatous diseases, consistent with expert diagnostic pathways.

(2)Specificity of Prednisone Treatment and Clarification on Concomitant Medications

①Specificity of Treatment and Concomitant Immunosuppressants: This study specifically aimed to analyze the effect of oral prednisone. To ensure a well-defined exposure group, we rigorously reviewed the database and applied explicit exclusion criteria (detailed in the Methods section), thereby excluding patients who received other corticosteroid formulations (e.g., local injections). While a minimal number of patients in the cohort had received other immunosuppressants such as methotrexate, the sample size was too small for separate analysis. The treatment exposure for the analyzed cohort is therefore specific to oral prednisone versus non-use.

②Recording of Other Concomitant Medications: Our database primarily captured medications relevant to the core treatment and pathological mechanism of GLM. Non-steroidal anti-inflammatory drugs (NSAIDs), used for symptomatic relief in this context, were not systematically recorded or analyzed in this study. We consider their role to be primarily symptomatic, with likely limited direct impact on the core inflammatory process of GLM or the long-term recurrence outcome which was our primary endpoint. In our primary statistical analyses, we rigorously controlled for known key potential confounders, such as antitubercular therapy, by including them as covariates in the multivariable Cox regression model (Table 1).

We have added clarifications on these points in the revised "Data and Samples" and "Statistical Analysis" sections. We believe this accurately addresses the reviewer's concerns regarding diagnostic rigor and the definition of the treatment group. We are grateful for these valuable comments, which have helped us present our methodology with greater precision.

**9. Please explain when were the lab tests done? As the test results were related to disease prognosis, the timing of the tests is very important. Usually, all GLM patients do not attend at the first disease presentation. Therefore, tests are taken at different disease stages. How was the condition of lab test timing in your database? If the mentioned concerns existed in your study (as expected), please mention this limitation in the discussion.**

Response:

We thank the reviewer for raising this important point. We have added a clarification regarding the collection and processing of laboratory data in the Methods section (under "Data Collection and Processing" or "Statistical Analysis").

We acknowledge that, due to the specificity of the disease, the tests were indeed performed at different stages of the disease. To establish a consistent analytical standard, we adopted the "peak value during the disease course" to reflect the disease activity level. This methodological approach and its rationale have now been explicitly described in the manuscript.

**10. Fig.1: Although well-organized and very useful, I suggest that sentences are shortened. Usually, short sentences or expressions are used in these types of workflows. E.g., instead of ‘614 pts with GLM were ultimately included in the analysis’, a shortened version as ’Finally included: 614 patients’ would be more suitable for the figure. This should be done in other parts of the figure also. Importantly, the ’It was hypothesized that …’ is not appropriate for the figure. It could be replaced by something like ’Assessment of prednisolone effects’.**

Response:

Thank you for this helpful suggestion. We agree that concise phrasing is more appropriate for a workflow figure. In the revised manuscript, the text in Figure 1 will be shortened to key phrases or bullet points (e.g., "Finally included 614 patients"), and the hypothesis statement will be replaced with a descriptive title such as "Assessment of Treatment Effects and Prognostic Factors."

**11. Line 144: How do authors explain the effect of the affected side on prognosis?**

Response:

We thank the reviewer for raising this interesting observation regarding the significant association between the affected side and prognosis in our multivariable analysis.

We fully acknowledge this statistically significant finding. The primary aim of the current study was specifically to investigate the impact of oral prednisone treatment protocols on recurrence risk in GLM. The inclusion of the affected side in the model served as a covariate for adjustment purposes. Given that the biological or clinical mechanisms underlying this association were not the focus of our pre-specified research objectives, and considering the retrospective nature of our data, we do not have a pathophysiological explanation readily available within the scope of this manuscript.

We agree with the reviewer that this association presents a compelling question for future research. We will explore this direction in subsequent studies. Thank you for this valuable suggestion.

**12. Tab 1: For prolactin, please provide the normal range. Also, define hyperlipidemia (which types of lipids? The ranges?). Was bromocriptine used for hyperprolactinemia? These should be described shortly in the footnote to make the table understandable and useful.**

Response:

We thank the reviewer for the valuable suggestions to improve the clarity of Table 1. We will address the points as follows in the revised manuscript:

(1)Prolactin Normal Range: As suggested, the reference range for serum prolactin in our laboratory (80.56–650.84 uIU/ml) will be clearly stated in the footnote of Table1.

(2)Definition of Hyperlipidemia: The specific diagnostic criteria for hyperlipidemia used in our study will be added to the Methods section. The condition was defined as meeting at least one of the following: total cholesterol ≥ 220 mg/dL, low-density lipoprotein cholesterol (LDL-C) ≥ 140 mg/dL, high-density lipoprotein cholesterol (HDL-C) < 40 mg/dL, or triglycerides ≥ 150 mg/dL.

(3)Indication for Bromocriptine Therapy: Regarding the reviewer’s question, bromocriptine was used in our cohort primarily in two contexts: (a) for patients with documented hyperprolactinemia (PRL > 650 µIU/mL), and (b) as an off-label adjuvant therapy for a subset of patients with normal PRL levels but intraoperative findings of ductal secretions. As the indication was not the focus of our study on oral prednisone, and to maintain clarity and conciseness, this detail is not elaborated within the manuscript itself.

We believe these modifications address the reviewer's comments and will enhance the transparency of our presentation. We appreciate the reviewer's attention to these details.

**13. Tab. 1: For the age at diagnosis, which carried a worse prognosis, younger or elder age?**

Response:

Thank you for your insightful question regarding the prognostic role of age at diagnosis, which prompted a re-evaluation of this result.

(1)Clarification of Analysis Results

In the univariable analysis, age at diagnosis did not show a statistically significant association with recurrence risk (P=0.126). However, in the multivariable Cox model (adjusted for confounders like affected side and treatment factors), age as a continuous variable reached significance (HR=0.940, 95% CI: 0.905-0.977, P=0.002). Crucially, the shift from non-significant in univariable to significant in multivariable analysis strongly suggests that age is not an independent prognostic driver. Its effect appears mediated through or dependent on other clinical variables in the model. Consequently, we have removed any description labeling age as an "independent prognostic factor" from the manuscript to prevent misinterpretation.

(2) Interpretation of the Hazard Ratio (HR)

The HR of 0.940 indicates that, with other model factors held constant, each one-year increase in age was associated with a ~6% lower recurrence risk in this cohort. This suggests a trend toward lower risk in older patients.

(3)Consideration of Confounding and Future Research

This observed association may stem from unmeasured confounding, such as age-related differences in treatment adherence, immune response, or disease subtype. As age was not a pre-specified study focus and its mechanism remains unclear, we have not discussed it in the main text to avoid overinterpreting a secondary finding. It remains an interesting observation for future hypothesis-driven research.

Thank you for highlighting this point, which has improved the precision of our results presentation.

**14. Lines 152-154: These descriptions should come in the Methods, not the results. Also, please explain what were the non-prednisone treatments. The overall treatment strategies are described in lines 97-100; however, we need to know, for example, if you had patients who only received antibiotics? Or only anti-tuberculosis agents? Or if bromocriptine was used alone, and regardless of the prolactin status? Regarding the higher recurrence in the prednisone group (as mentioned in lines 154-156 and Fig 2), it is crucial to know about the comparison group details.**

Response:

Thank you for these valuable suggestions, which are crucial for improving the clarity of our methodology and the interpretability of our results. We have revised the manuscript accordingly, addressing each of your points as follows:

(1)Relocation of the Grouping Description

We have moved the description of patient grouping from the Results section (original Lines 152-154) to the "Statistical Analysis" subsection within the Methods. The group definitions are now clearly stated when the analytical strategy is first introduced.

(2)Detailed Description of the Non-Prednisone Treatment Group

You correctly pointed out that understanding the composition of the comparison group is essential for interpreting the results. In clinical practice, the management of GLM typically involves combination therapy, which is also reflected in the treatment data of our study. To provide complete information, all treatment categories received by patients in both the oral prednisone and non-prednisone groups, along with their specific distributions, have been detailed in the supplementary table (S2 Table) comparing the baseline characteristics of the two groups for your review.

(3)Ensuring Comparability Between Groups and Acknowledging Methodological Limitations

To directly address your concern regarding the validity of comparing recurrence rates, we controlled for therapeutic heterogeneity through two methods:

①Multivariable Cox Model: In Table 1, the effect of oral prednisone (yes/no) was adjusted for key covariates including antitubercular therapy, bromocriptine therapy, abscess drainage, and surgical excision. This statistically controls for the influence of these other treatments.

②Propensity Score Matching (PSM): We performed 1:1 PSM, balancing all available baseline characteristics and prior treatments (including the specific pharmacological and surgical interventions listed above) between the matched prednisone and non-prednisone patients. The persistent finding of a higher recurrence risk in the prednisone group within this matched cohort supports the robustness of the association.

③Acknowledgment of PSM Limitations: We fully recognize that while PSM balances observed confounders, it cannot account for unmeasured or residual confounding factors. We have stated this point in the Limitations section of the Discussion.

These revisions enhance the transparency of our study design and ensure a more prudent presentation of the results. We are very grateful for your insightful feedback.

**15. Lines 283-285: The point is that the two groups: prednisone and non=prednisone are not compared regarding disease severity, so the results might just mirror the disease severity. Please explain if that is not the point, and otherwisw discuss the point as such and add it in the limitations.**

Response:

Thank you for raising this core concern. You are correct to point out that differences in disease severity between treatment groups in a non-randomized study are a critical issue that must be rigorously scrutinized when interpreting results. We fully agree and have employed multiple strategies in our study design and analysis to address this challenge as thoroughly as possible, while explicitly acknowledging its limitations in the Discussion.

Our main response integrates our previous detailed replies to related comments and external evidence supporting our findings, as outlined below:

(1)Statistical Adjustment for Disease Severity: In our primary multivariable Cox proportional hazards model (Table 1), we adjusted for a series of objective, clinically used proxies for disease severity that directly inform the decision to initiate prednisone. These included: the presence of a mammary abscess, maximum lesion diameter (≥4 cm), multiple/bilateral lesions, and elevated white blood cell count (≥10×10⁹/L). After adjusting for these measurable severity indicators, oral prednisone use remained a strong and independent predictor of recurrence (HR = 2.534, 95% CI: 1.615–3.975, P < 0.001).

(2)Balancing Groups via Propensity Score Matching (PSM): We further performed 1:1 PSM. In the matched cohort (171 pairs), we successfully balanced all observable baseline characteristics between the prednisone and non-prednisone groups, including the aforementioned severity indicators and other treatments (e.g., antibiotics, antitubercular therapy, surgery). In this matched set, the prednisone group still showed a significantly higher 1-year recurrence rate (21.8% vs. 13.6%, P=0.042), providing additional support for the association between prednisone exposure and recurrence risk.

(3)Explicit Acknowledgement of Residual Confounding as a Core Limitation: We fully recognize that, despite these measures, the influence of unmeasured or residual confounding factors (including the lack of a gold-standard composite severity score) cannot be completely ruled out. This is an inherent limitation of observational research. We have emphasized this point in the Limitations section of the Discussion, explicitly stating: "Specifically, while propensity score matching was employed to balance observed confounders including concomitant therapies, it cannot account for unmeasured or residual confounding factors."

(4)External Research Support for the Finding: It is important to note that our observation is not isolated. Our 1-year recurrence rate (29.4% in the prednisone group) aligns with the wide range (24.7% to 46.9%) reported in multiple previous studies. More importantly, several studies and a 2024 meta-analysis of 65 studies have reported a higher recurrence rate with oral steroid therapy compared to observation alone [16,23,25], with rates of 24% and 11%, respectively, in that meta-analysis. These consistent findings across studies, combined with the results from our large-scale cohort (n=614, exceeding most previous studies), collectively strengthen the observed association.

Summary: We did address potential differences in disease severity between the groups by statistically adjusting for and matching on the severity indicators we could measure. The possibility that "the results might just mirror the disease severity," which you rightly highlight, is precisely what we sought to mitigate through these analyses and have transparently discussed as a core limitation. These points are fully addressed in the revised manuscript.

We believe this approach achieves a high degree of methodological rigor within the framework of observational data and maintains the necessary caution in interpreting the results. We thank the reviewer again for prompting us to articulate this critical point more clearly.

**16. Lines 309-327: These are hypothetical reasons for the higher recurrence in patients with early-onset treatment, and they do not follow a very sound rational. Patients who attended later probably had a less severe disease, and those that attended earlier had a more severe disease. So, treatment was begun sooner, but recurrence was higher. Although this is not certain, but it should be mentioned as a probable reason for these results, and recognized as a main confounding factor in the discussion and limitations.**

Response:

We sincerely thank the reviewer for this critical and insightful comment. The reviewer is absolutely correct to point out that our original discussion did not sufficiently prioritize the most straightforward and clinically relevant explanation for the observed association—namely, confounding by baseline disease severity. We agree that the interpretation of our findings must first and foremost address this key issue of “confounding by indication.”

In direct response to the reviewer's suggestion, we have fundamentally restructured the relevant paragraph in the Discussion section. The revised text now explicitly and upfront presents “confounding by disease severity” as the primary alternative explanation for the higher recurrence rate observed in the early-treatment group. We clarify that patients requiring treatment within 6 weeks likely had more severe disease at baseline, and that this intrinsic disease burden could be a major driver of the observed outcome, independent of the timing of treatment initiation. We also acknowledge that while our models adjusted for measurable severity proxies, residual confounding remains a key limitation.

Subsequently, and in a more cautious and complementary manner, we then discuss the potential pathophysiological mechanisms (e.g., steroid tapering rebound, natural disease course filtering) that could be explored if an independent effect of treatment timing is confirmed in future studies designed to better control for disease severity.

We believe this revision has significantly strengthened the logical rigor and scientific balance of our discussion by directly addressing the reviewer’s valid concern. The changes ensure that our interpretation aligns with the fundamental principles of causal inference in observational research. We are grateful for this valuable feedback, which has greatly improved the quality of our manuscript.

**17. Lines 328-331: Treatment duration depends on the disease response to treatment, so medicines cannot be cut before obtaining an acceptable response. Do the authors mean to discontinue steroids while the disease is improving but we are still waiting for recovery? The reason for the lower recurrence with a shorter treatment duration might simply be because of less severe disease. Authors should consider this important point in the discussion and limitations.**

Response:

We thank the reviewer for this important insight, which further refines the interpretation of our findings regarding treatment duration. We fully agree that the duration of therapy is intrinsically linked to, and largely determined by, the underlying disease severity and its response to treatment, rather than being an independent causative factor.

In our original manuscript, we had already highlighted “disease severity” as the primary explanation for discrepancies in findings related to treatment duration. In direct response to the reviewer’s suggestion to make this link more explicit and dynamic, we have revised the corresponding sentence in the Discussion to emphasize that course length is guided by “disease severity and treatment response” and have added the clarifying statement: “Thus, treatment duration may act more as a marker of disease behavior than as an independent modifiable factor.”

Most importantly, the methodological limitation inherent in this interpretation—that treatment duration is subject to confounding by indication—is already explicitly acknowledged in the first point of our study’s Limitations section, which states: “…introduces the potential for unmeasured confounding (e.g., confounding by indication for prednisone use)…”

We believe that with this targeted reinforcement in the Discussion, alongside the pre-existing acknowledgment in the Limitations, the reviewer’s valid concern has been adequately addressed. We appreciate this comment, which has helped us sharpen the clinical logic of our discussion.

**18. Lines 332 to 358: This part should be shortened and simplified with an accurate wording. Authors should pay attention to the tense of the verbs and the use of ‘this’ and ‘that’ for theirs vs. other studies.**

Response:

Thank you for pointing out the need to improve the wording and clarity in this part of the discussion. We have revised the entire text based on your suggestions.

We focused on making the text clearer and more direct. We shortened some sentences, made sure the verb tenses are consistent (using past tense for our findings and present for general statements), and clarified the references so that it's always clear when we are talking about "our study" versus "other studies" (like the one by Wang et al.).

We also fixed a couple of small wording issues and tried to make the logic flow more smoothly. We believe the revised version is now much easier to follow.

Thanks again for the helpful feedback.

**19. Lines 367-369: Lower doses are most probably due to less severe disease, which explains the lower recurrence. As the clinical signs are not compared between the two groups, we cannot know whether the low and high dose groups were similar in this regard, and they were most probably not. This also applies to the weight-adjusted dose. This key point should be considered and alluded to in the discussion and limitations.**

Response:

Thank you for raising this critical point regarding the interpretation of the dose-outcome relationship. We agree that the potential for confounding by disease severity is a key consideration when comparing different dose groups, as clinicians may naturally prescribe lower doses to patients with milder presentations.

In direct response to your suggestion, we have revised the discussion in the “Dosage” subsection (specifically in the paragraph following the identification of the 0.4 mg/kg/day cutoff). We have inserted a new statement that explicitly acknowledges this alternative explanation, noting that “the prescribed dose may be correlated with disease severity at baseline” and that this possibility “represents a key consideration for the results, also applying to the weight-adjusted dose analysis.”

Furthermore, we wish to highlight that this methodological caution aligns with the first limitation already stated in our manuscript: “...introduces the potential for unmeasured confounding (e.g., confounding by indication for prednisone use)...” The point you raised is a specific and important instance of this general limitation, which we have now addressed both in the overarching limitations section and within the specific context of the dosage analysis.

We believe these revisions provide a more balanced and rigorous discussion of our findings. Thank you for this insightful comment.

**20. Apparently, the results and discussion indicate that the non-prednisone treatments, the lower doses of prednisone, the later-onset treatment initiation, and the shorter duration of prednisolone use is either better that or not different with the comparison group better. Wouldn’t all these reflect that prednisolone is better not used?**

Response:

Thank you for your careful review and valuable comments on our manuscript. You raised the point that the results indicate non-prednisone treatments, lower doses of prednisone, later treatment initiation, and shorter treatment durations appear to be either better than or not different from the comparison group. This understandably leads to the question of whether prednisone should be avoided altogether. We appreciate this important observation and would like to provide further clarification regarding the intent and clinical context of our study.

Prednisone (or prednisolone) continues to play a significant role in the management of granulomatous lobular mastitis (GLM), particularly during the acute phase, primarily due to its ability to rapidly alleviate inflammation and pain and improve patient symptoms. Current guidelines also recommend its use under certain conditions. However, the aim of our study was not to completely negate the use of prednisone, but rather to explore, while acknowledging its clinical value, how to use this drug more safely and rationally to balance its therapeutic benefits against potential risks, such as increased relapse rates.

Our data analysis suggests that prednisone use is associated with a higher risk of relapse, and that the timing of treatment initiation and the dosage may be key factors influencing outcomes. Therefore, we are not advocating for the complete abandonment of prednisone. Instead, we propose a more cautious strategy in clinical practice—for instance, initiating treatment after a 6-week symptom interval and using a lower dose (≤ 0.4 mg/kg/day)—with the goal of controlling symptoms while potentially minimizing the risk of recurrence.

We have emphasized in the Discussion and Conclusion sections that the use of prednisone should be individualized, weighing the benefits and risks according to each patient's specific situation. As this is an observational study, the proposed treatment strategy requires further validation through future prospective research.

Thank you again for prompting us to clarify the stance of our study more explicitly. We have refined the relevant sections of the manuscript to more clearly convey the core message of "optimizing use rather than completely rejecting" prednisone in GLM management.

**21. Overall, as disease severity has not been considered in the comparison between groups, results should be taken cautiously. The very positive sentences that present results of this study as doubtless data need to be re-written to reflect the uncertainty of the mentioned points. The limitations also should be revised accordingly.**

Response:

Thank you for raising this essential point regarding disease severity and the cautious interpretation of our results. We fully agree with your assessment. The potential for confounding by differential disease severity between comparison groups is a fundamental limitation in observational studies like ours, and it necessitates a careful, nuanced presentation of the findings.

We have carefully revised the manuscript to address your specific concerns as follows:

(1)Tone and Wording of Results/Discussion:

We have systematically reviewed the Results and Discussion sections and toned down any language that may have presented the findings as overly definitive. Phrases suggesting causation have been replaced with more cautious language indicating association, correlation, or observation (e.g., "was associated with," "may suggest," "our data indicate"). We have added qualifying statements to emphasize that the results are derived from an observational analysis and are subject to the limitations inherent in such a design.

(2) Revised and Strengthened Limitations Section:

We have substantially expanded and refocused the "Limitations" subsection in the Discussion to directly and prominently address the issue you highlighted. The revised text now explicitly states:

The lack of randomization means that differences in baseline disease severity between the treatment groups could be a significant confounding factor influencing the observed outcomes (confounding by indication).

While we employed statistical methods (multivariable adjustment and propensity score matching) to control for measurable proxies of disease severity (e.g., abscess presence, lesion size, bilateral involvement, leukocytosis), these may not fully capture the complete clinical spectrum of disease severity or activity.

Therefore, the associations we report, particularly regarding treatment timing and dosage, should be interpreted with caution. They may reflect the underlying disease phenotype (more aggressive disease prompting earlier/higher-dose treatment) rather than a direct, modifiable effect of the treatment parameters themselves.

Consequently, our findings are hypothesis-generating and must be validated in future studies designed to more rigorously assess and control for baseline disease severity.

(3) Connection to Previous Analyses:

We wish to clarify that the concern regarding disease severity is precisely what we sought to mitigate through the analytical methods described in our responses to previous comments (Points 15, 16, 17, and 19). The multivariable models and propensity score matching were implemented specifically to address this type of confounding. However, as we now more clearly acknowledge, these methods cannot eliminate all residual confounding.

Your comment has been invaluable in ensuring our manuscript accurately reflects the inherent uncertainties of the research and avoids overstating the conclusions. We believe the revised text now presents a more balanced and scientifically rigorous interpretation of our data.

Thank you again for this critical feedback, which has significantly improved the quality of our manuscript.

Best regards,

Luo Jing
